# Supplementary material for: ARID1A Deficiency Regulates Anti-Tumor Immune Response in Esophageal Adenocarcinoma
Source: Cancers (Basel). 2023 Nov 12;15(22):5377. doi: 10.3390/cancers15225377 (PMC10670331; doi:10.3390/cancers15225377)
Supplement: Supplementary file 1 [file cancers-15-05377-s001.zip › cancers-2693710-supplementary.pdf]

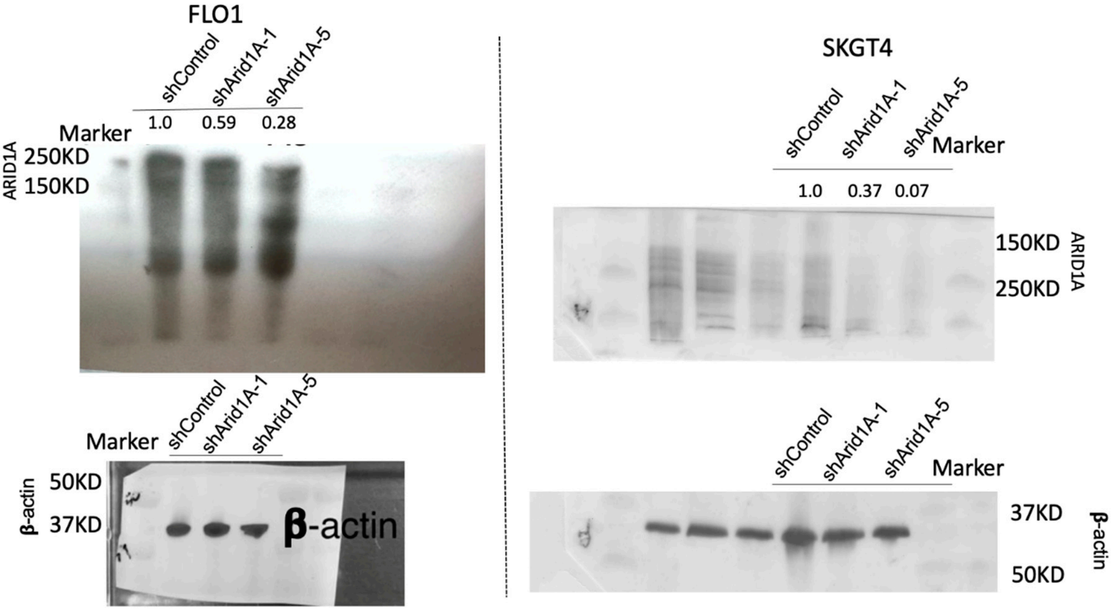


**Supplement Figure S1**. Western-blot of ARID1A in FLO1 and SKGT4 ARID1A-knockdown cells by shRNA.

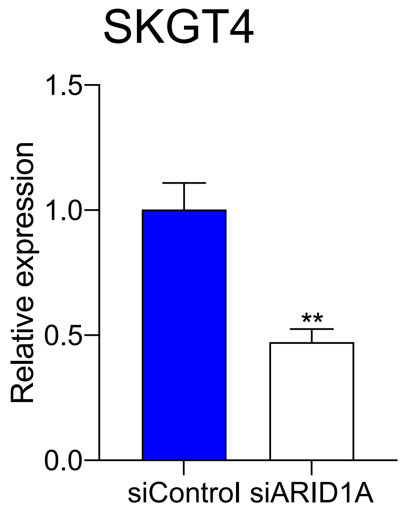


**Supplement Figure S2**. The mRNA expression of ARID1A upon knockdown by siRNA in SKGT4 cells.

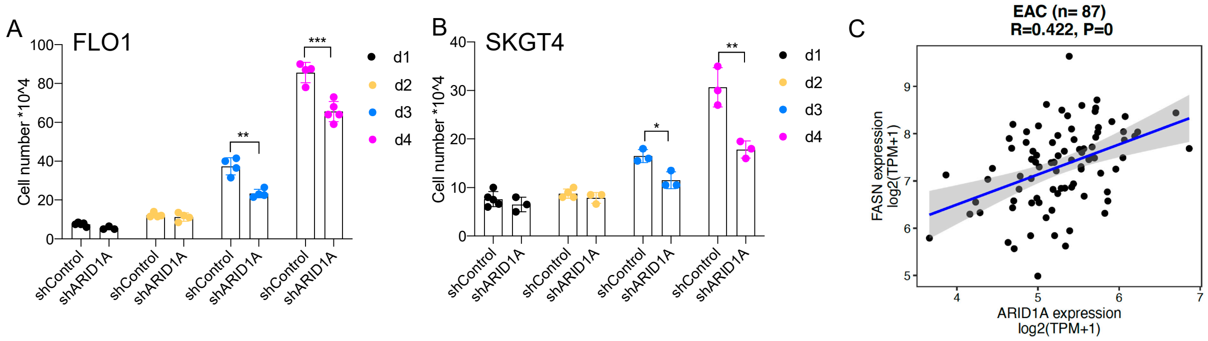


**Supplement Figure S3.** ARID1A knockdown inhibit cell proliferation in EAC. A-B) Cell proliferation of ARID1A knockdown and control by shRNA in A) FLO1 and B) SKGT4 from day 1 to day 4. C) The expression correlation between ARID1A and FASN in EAC based on TCGA data. Paired, 2-tailed $t$ test: * $p < 0.05$, ** $p < 0.01$, *** $p < 0.001$.

**Supplement Table S1.** Real-time PCR Oligonucleotide Sequence.

| Gene | Forward Primer Sequence | Reverse Primer Sequence |
|---|---|---|
| ***Human*** | | |
| **TBP** | TGTATCCACAGTGAATCTTGGTTG | GGTTCGTGGCTCTCTTATCCTC |
| **ARID1A** | AAGCCACCAACTCCAGCATCCA | CGCTTCTGGAATGTGGAGTCAC |
| **B2M** | CCACTGAAAAAGATGAGTATGCCT | CCAATCCAAATGCGGCATCTTCA |
| **BATF2** | CACCAGCAGCACGAGTCTC | TGTGCGAGGCAAACAGGAG |
| **BST2** | CACACTGTGATGGCCCTAATG | GTCCGCGATTCTCACGCTT |
| **CLL2** | AGAATCACCAGCAGCAAGTGTCC | TCCTGAACCCACTTCTGCTTGG |
| **CXCL9** | CTGTTCCTGCATCAGCACCAAC | TGAACTCCATTCTTCAGTGTAGCA |
| **CXCL10** | GGTGAGAAGAGATGTCTGAATCC | GTCCATCCTTGGAAGCACTGCA |
| **CTSS** | TGGATCACCACTGGCATCTCTG | GCTCCAGGTTGTGAAGCATCAC |
| **HSPA1A** | GGCCTTGAGGACTTTGGGTTA | TGGGAATGCAAAGCACACG |
| **IFITM2** | ATGAACCACATTGTGCAAACCT | CGGAGTAGGCGAATGCTATGAA |
| **IFITM3** | ATGTCGTCTGGTCCCTGTTC | GTCATGAGGATGCCCAGAAT |
| **IFI44** | TTTTCGATGCGAAGATTCACTGG | CCTGATGCGTTACATGCCCTT |
| **IFI44L** | AGCCGTCAGGGATGTACTATAAC | AGGGAATCATTTGGCTCTGTAGA |
| **IRF2** | CATGCGGCTAGACATGGGTG | GCTTTCCTGTATGGATTGCCC |
| **IRF7** | GCTGGACGTGACCATCATGTA | GGGCCGTATAGGAACGTGC |
| **OASL** | CCATTGTGCCTGCCTACAGAG | CTTCAGCTTAGTTGGCCGATG |
| **STAT3** | ACCAGCAGTATAGCCGCTTC | GCCACAATCCGGGCAATCT |
| **PSMB8** | CCTTACCTGCTTGGCACCATGT | TTGGAGGCTGCCGACACTGAAA |
| **PSMB9** | CGAGAGGACTTGTCTGCACATC | CACCAATGGCAAAAGGCTGTCG |
| **PSMB10** | GGACAAGAGCTGCGAGAAGATC | ATCTTGGACGCCACCATCCGTG |
| **FASN** | CCGAGACACTCGTGGGCTA | CTTCAGCAGGACATTGATGCC |
| **SREBF1** | GCCCCTGTAACGACCACTG | CAGCGAGTCTGCCTTGATG |
| **MVD** | TGAAGGACAGCAACCAGTTC | CAGGAGATGGCATTGAGGTAAG |
| **SCD1** | GCCCCTCTACTTGGAAGACGA | AAGTGATCCCATACAGGGCTC |
| **SREBF2** | AACGGTCATTCACCCAGGTC | GGCTGAAGAATAGGAGTTGCC |
| ***Mouse*** | | |
| **TBP** | CTACCGTGAATCTTGGCTGTAAAC | AATCAACGCAGTTGTCCGTGGC |
| **ARID1A** | GACCCCTCAGTCATCCAGTC | GAGTATGGGTTAGTCCCACCA |
| **B2M** | ACAGTTCCACCCGCCTCACATT | TAGAAAGACCAGTCCTTGCTGAAG |
| **BST2** | TGTTCGGGGTTACCTTAGTCA | ACCCGTCTCTACAGGCCAC |
| **CCL2** | GCTACAAGAGGATCACCAGCAG | GTCTGGACCCATTCCTTCTTGG |
| **CD74** | AGATGCGGATGGCTACTCC | TCATGTTGCCGTACTTGGTAAC |
| **CXCL9** | CCTAGTGATAAGGAATGCACGATG | CTAGGCAGGTTTGATCTCCGTTC |
| **CXCL10** | ATCATCCCTGCGAGCCTATCCT | GACCTTTTTTGGCTAAACGCTTTC |
| **CTSS** | GCATAGAGGCAGACGCTTCCTA | CCACTGCTTCTTTCAGGGCATC |
| **HSPA1A** | TGGTGCAGTCCGACATGAAG | GCTGAGAGTCGTTGAAGTAGGC |
| **PSMB8** | CCTTACCTGCTTGGCACCATGT | TTGGATGCTGCAGACACGGAGA |
| **PSMB9** | TACCGTGAGGACTTGTTAGCGC | GGCTGTCGAATTAGCATCCCTC |

| | | |
|---|---|---|
| **PSMB10** | TTCGGGTTCCTCATGCACGCAA | TTTGTCCGCCACAACCGAATCG |
| **FASN** | AGGTGGTGATAGCCGGTATGT | TGGGTAATCCATAGAGCCCAG |
| **SCD1** | TTCTTGCGATACACTCTGGTGC | CGGGATTGAATGTTCTTGTCGT |
